# EMI Shielding Nanocomposite Laminates with High Temperature Resistance, Hydrophobicity and Anticorrosion Properties

**DOI:** 10.3390/nano11113155

**Published:** 2021-11-22

**Authors:** Shaojun Wu, Zhiyong Zhao, Hongliang Hou, Xiang Xue

**Affiliations:** 1 School of Materials Science and Engineering, Harbin Institute of Technology, Harbin 150001, China; wusj0303@163.com; 2 AVIC Manufacturing Technology Institute, Beijing 100024, China; 3 School of Mechanical, Electrical and Information Engineering, Shandong University (Weihai), Weihai 264209, China; zhaozhy@sdu.edu.cn

**Keywords:** composite prepreg, EMI shielding, high temperature resistance, hydrophobicity, corrosion resistance

## Abstract

High-performance multifunctional EMI shielding composite fabricated by low-cost method is increasingly required. Herein, novel EMI shielding nanocomposite laminates, consisting of composite prepreg of carbon fiber/epoxy resin/carbon nanotube film, were manufactured by facile electric heating of carbon nanotube film. The results indicated that composite with excellent specific shielding effectiveness of 0.07 dB/μm, 47 dB cm^3^/g and metamaterial properties can be designed by composite prepreg, and the primary shielding mechanism of it was reflection loss, along with interface polarization loss and conductive loss, which was superior to lots of shielding materials including carbon nanotube-based, carbon black-based, carbon nanofiber-based and graphene-based materials reported previously. Meanwhile, highly required excellent properties, including the thermostability with initial decomposition temperature up to 300 °C, hydrophobicity over contact angle of 115°, corrosion resistance of the composite with metal-free modification, and function as structural laminate compared with previous studies were demonstrated, which suggested tremendous potentials of the multifunctional EMI shielding composites in harsh environment.

## 1. Introduction

With the widespread use of portable electronic devices and wearable devices, there is an increasing need for efficient, low-cost and multifunctional EMI shielding materials. Carbon nanotubes (CNTs) offer substantial advantages over traditional metal EMI shielding materials in this respect due to their excellent electrical conductivity, mechanical strength, thermal stability and low density [1], arousing strong interest in it as conductive fillers of EMI shielding materials recently. For example, shielding composites with CNT powder and polymer matrix, such as ultra-high molecular weight polyethylene [2], epoxy resin [3], cellulose [4], natural rubber [5], polystyrene [6], polymethyl methacrylate [7], polycarbonate [8], etc., have been fabricated by solution process, dry mixing or melt mixing etc., however, the poor quality of the dispersion and lower carbon nanotube contents are the major limitation for applications of CNTs/polymer composites as shielding materials. Carbon nanotube film (CNTF) embedded various resin matrices could provide a way out of the dilemma, and this way avoided the problem of poor dispersion. Thus, several representative works have been performed as well. For instance, the EMI shielding composites containing CNTF and various resin matrices were reported by vacuum bagging process, bonding, hot-melt method or spin-coating, etc. [9,10], revealing excellent EMI shielding properties.

Despite a series of mentioned-above studies on shielding composites with CNTF and polymers, the function of them was usually single, which is problematic when meeting complex actual scenarios, especially EMI shielding materials with high temperature resistance, hydrophobicity and metal-free modification anticorrosion are highly desirable [11,12,13]. Inspired by recent reports about nanostructures of polypyrrole and the relevant carbonized analogs with excellent EMI shielding properties, thermal stability, resistivity against ageing and energy storage property [14,15], herein, to fulfill the ever-growing demands, we intended to manufacture multifunctional EMI shielding nanocomposite laminate (CEC materials) by composite prepreg of carbon fiber-epoxy-CNTF (CF-EP-CNTF, CEC) to investigate aforementioned multi-properties of the materials fabricated by means of CNTF electric heating (e-heating), which recently has been known as an efficient and advanced heating way with heat transfer efficiency of over 90% [16,17], and preparations of CEC materials by traditional ways of oven and hot-press were conducted for comparison simultaneously. Mainly, the multifunctional properties, including EMI shielding, thermostability, self-cleaning and anticorrosion of material without metal modification, were analyzed by regulating stacking number, stacking angle of composite prepregs and areal density of CNTF in composite prepreg, results indicated that this multifunctional CEC material with good specific shielding effectiveness and properties of metamaterial can be obtained by an efficient and simple CNTF e-heating method.

## 2. Material and Methods

### 2.1. Fabrication of Composites

**Figure 1:**
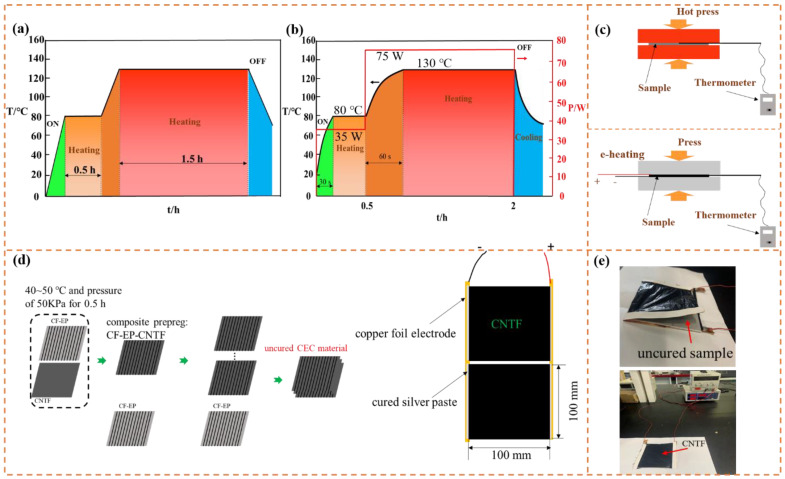
The curing temperature profile of samples by (**a**) oven and hot press, (**b**) e-heating; schematic diagram of (**c**) hot press (up) and e-heating (down) and (**d**) composite prepreg, sample preparation method and CNTF electric heater; (**e**) images of sample.

CNTFs were made using floating catalyst chemistry vapor deposit (FCCVD) method, which was detailed in a previous publication [18]. CF prepreg used in composites was supplied by ZhongFu Carbon Fiber Products Co. Ltd., Yixing, China. The CF prepreg consisted of 53 wt % unidirectional T300 carbon fiber and 47 wt % epoxy resin matrices. Composite prepreg CF-EP-CNTF was fabricated by compressing CF prepreg and CNTF at condition of 40~50 °C and 50 KPa for 0.5 h, the uncured samples with size of 100 × 100 mm^2^ were stacked up along one direction by hand and CNTF heater for CEC composites fabrication was prepared as shown in Figure 1d. Then the uncured sample are covered with a layer of high temperature resistant cellophane and subsequently two surfaces of it were covered by CNTF heater as shown in Figure 1e, wherein the high temperature resistant cellophane could benefit the separation of cured sample and CNTF heater. Lastly the e-heating process of uncured sample was carried out by CNTF and DC power supply (ATTEN, Antaixin Technology Co., Ltd., Shenzhen, China), and a lower pressure of ~50 KPa was supplied simultaneously shown in Figure 1c. To evaluate EMI shielding properties of CEC materials prepared by CNTF e-heating, control samples were also made by curing hybrid laminate of 1CF-EP-CNTF/1CF-EP via oven (DZF, Kewei Yongxing Instrument Co., Ltd., Beijing, China) and hot press (JC-1, Jiangchuan Printing Equipment Co., Ltd., Guangzhou, China) with curing process displayed in Figure 1a,c. A higher pressure of ~0.5 MPa was supplied while hot press was performed according to previous publication [9]. The CEC samples were obtained after curing process was finished and monitored temperature cooled to room temperature. Detailed presentations of the obtained CEC materials denoted as sample 1–15 were exhibited in Table 1.

### 2.2. Characterization

To detect the curing degree of the CEC composites, uncured sample was tested by differential scanning calorimetry (DSC) (DSC404F3, Netzch, Germany) at a scanning rate of 5 °C/min from 25 °C to 250 °C to calculate the total heat of the complete reaction (H_T_), the residual reaction heat (H_r_) for the sample cut from the cured sample was thus obtained by same test procedure. The curing degree of the CEC composites was then calculated through the formula: (H_T_ − H_r_)/H_T_ × 100%. The electrical resistance of the CNTF was detected using a four-probe meter (H7756, Heng’aode Technology Co., Beijing, China), and then conductivity σ of it was calculated by formula: σ = 1/(Rt), where R, t denote electrical resistance and thickness, respectively. The cross-section morphology of CEC samples was characterized by optical microscope (CEWEI, Cewei Technology Co., Ltd., Beijing, China). 

In order to evaluate the shielding properties of the samples, all samples were cut into rectangle block with size of 22.9 × 10.2 mm^2^, the EMI SE, permittivity and permeability were measured using a vector network analyzer (VNA, PNA-N5244A, Agilent Technology Company, Santa Clara, CA, USA) in the frequency range of 8.2–12.4 GHz by the waveguide methods. The detailed SE measurement technique used the S-parameters obtained from VNA to evaluate the total shielding effectiveness (*SE_T_*), as:

$$SE_{T}=10lg\frac{1}{S_{21}^{2}}=10lg\frac{1}{S_{12}^{2}}$$

where *S*_21_ and *S*_12_ are the forward and reverse transmitted coefficients respectively. The *SE_T_* has contributions from reflection loss (*SE_R_*) and absorption loss (*SE_A_*) and they are given by:

$$SE_{R}=10lg\frac{1}{1-S_{11}^{2}}=10lg\frac{1}{1-S_{22}^{2}}$$

$$SE_{A}=10lg\frac{1-S_{11}^{2}}{S_{21}^{2}}=10lg\frac{1-S_{22}^{2}}{S_{12}^{2}}$$

where *S*_11_ and *S*_22_ stand for the forward and reverse reflected coefficients [19].

Thermal stability and hydrophobicity of the CEC materials in air were investigated by thermogravimetric analysis (TG-DTG, STA-8000, PerkinElmer, Branford, CT, USA) and contact angle meter (FCA500B, Aifeisi Precision Instrument Co., Ltd., Shanghai, China), respectively. To determine the pH variation of aqueous hydrochloric acid solution and aqueous ammonia with different concentrations, which were employed to investigate the acid and alkali resistance of samples, litmus paper with the standard colorimetric card was used. 

## 3. Results and Discussion

### 3.1. Morphology and DSC Analysis

The cross-section microscope images of samples 1, 2 and 4 were revealed in Supplementary Materials Figure S1, which were cured by means of oven, hot press and CNTF e-heating, respectively. The void-free layered structure of samples 2 and 4 can be clearly observed, while the micropore can be found in sample 1 as shown in Supplementary Materials Figure S1a, indicating that the hot press and e-heating are efficient fabrication methods compared to oven in terms of structural integrity. Furthermore, according to insets in Figure S1a–c, hot press and e-heating could facilitate the combination of CNTF and epoxy resin with excellent liquidity, where sample 2 without obvious interface was displayed, while sample 4 showed vaguer interface of CNTF-resin in contrast to sample 1. The main cause for the microstructure difference was that pressure could aggregate flow of resin matrix during heating and eliminated the existence of hole in composites. Supplementary Materials Figures S2 and S3 present the cross-section morphology of representative samples with the same CNTF but a different structure, and the same structure but different CNTFs, respectively. The representative samples, such as samples 3, 4, 5 and 7, showed similar characteristics of void-free layers and vaguer interface as shown in Figure Supplementary Figure S2a–c, except sample 7 with obvious interface between two composite prepregs due to weaker interaction of two CNTF surfaces of CEC and absence of penetrated resin shown in Supplementary Materials Figure S2d. Supplementary Materials Figure S3 indicated that analogical cross-section morphology of 1CF-EP-CNT/1CF-EP with CNTF areal density regulated from 5.48 to 24.8 g/m^2^ can be obtained. 

To evaluate curing degree of the as-prepared CEC samples, thermal analysis was performed by DSC as shown in Supplementary Materials Figure S4, and the calculated results of curing degree were summarized in Supplementary Materials Table S1. The exothermic peak in DSC curves indicated that the crosslinking reaction of uncured pristine prepreg would occur at approximately 130 °C while DSC curves without exothermic peak were exhibited during heating process for sample 1–10, and curing degree of them surpassed 99%, revealing similar curing effect of the materials fabricated by e-heating and traditional methods.

### 3.2. EMI Shielding Properties 

**Figure 2:**
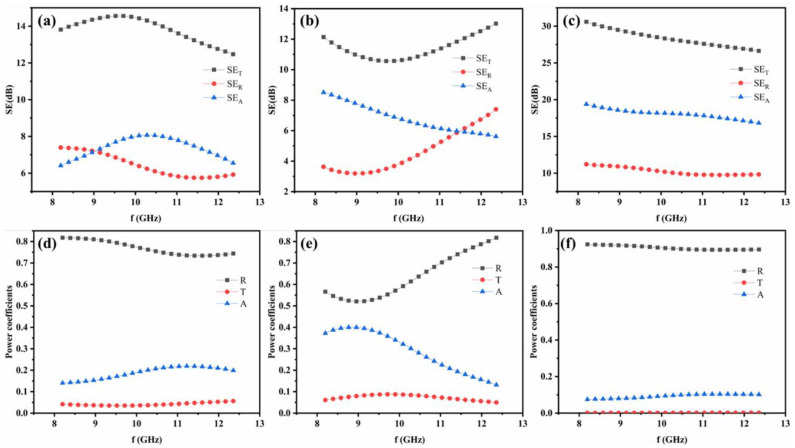
EMI SE and power coefficients R, T, A of (**a**,**d**) sample 1; (**b**,**e**) sample 2 and (**c**,**f**) sample 4 with same structure of 1CF-EP-CNTF/1CF-EP with areal density of 6.8 g/m^2^.

The EMI shielding properties evaluation of the samples cured by oven, hot press and e-heating were conducted firstly and showed as following. The EMI SE values of the samples 1, 2, and 4 were measured in the frequency range of 8.2–12.4 GHz, which is widely used in communication applications, as shown in Figure 2a–c. The observations indicate that the SE of sample 4 cured by CNTF e-heating outperform that of sample 1 and 2 cured by oven and hot press, respectively, which could result from the difference of microstructure portrayed in Figure S1. The micro holes in sample 1 would lead to damage of conductive net to some degree, much resin would penetrate into CNTF in composite prepreg when higher pressure of ~0.5 MPa was supplied for sample 2, leading to poor conductivity. However, sample 4 heated by e-heating at a lower pressure would avoid the micro holes and much resin penetration, while better combination between interfaces can be observed, resulting in less variation of conductivity of CNTFs. CEC composite with higher conductivity could cause higher SE on account of the proportional relation of SE and conductivity. To further analyze the EMI shielding mechanism of the as-fabricated CEC composites, the corresponding power coefficients of reflectivity (R), absorptivity (A), and transmission coefficient (T) were employed to evaluate the power balance of electromagnetic waves interacting with materials. As depicted in Figure 2, R is always higher than A although *SE_R_* is smaller than *SE_A_* regardless of the preparation methods, implying primary shielding mechanism of reflection loss [20,21,22,23]. 

**Figure 3:**
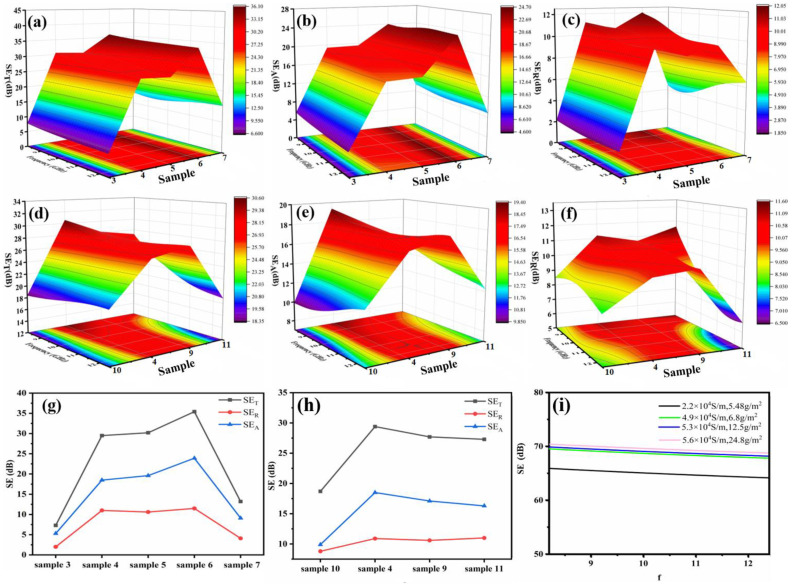
Three-dimensional presentation of (**a**) *SE_T_*, (**b**) *SE_A_*, (**c**) *SE_R_* of sample 3–7; three-dimensional presentation of (**d**) *SE_T_*, (**e**) *SE_A_*, (**f**) *SE_R_* of sample 9, 4, 8, 10; (**g**,**h**) trend of variation of SE at 9 GHz of samples; (**i**) fitted lines of CNTFs with different areal density by Formula (1).

To verify the influence of structures on the EMI shielding properties of the CEC materials, EMI shielding properties of materials manufactured by CNTF e-heating containing differing numbers of composite prepregs and CNTFs were investigated. Figure 3a–c exhibited the trend of variation of SE in the range of 8.2–12.4 GHz for sample 3–7 in three-dimensional presentation, displaying peak of SE among the samples. As we can see from Figure 3g, the SE at 9 GHz would increase from 7 dB of sample 3 to 36 dB of sample 6 by replacing CF-EP with CEC and growing number of the CEC, which was attributable to enhancement of total conductivity of composites with increased contents of conductive CNTFs. However, unsatisfying EMI SE of approximately 13 dB of sample 7 with two composite prepreg CECs was obtained, this occurrence could attribute to the poor combination of two CNTFs between composite prepregs due to the lack of resin penetration as shown in Supplementary Materials Figure S2d, probably leading to higher contact resistance. As far as the propagation characteristics of electromagnetic wave are concerned, electromagnetic wave would basically not pass through the CEC shielding materials due to transmission coefficient T of about zero shown in Figure S5, indicating that electronic devices would not be interfered by external electromagnetic wave while the designed composite was used as structural laminate. 

Figure 3d–f reveals the three-dimensional variation trend of EMI SE in X-band when the areal density of CNTFs in composite prepreg regulated from 5.48, 6.8, 12.5 to 24.8 g/m^2^ for samples with structure of 1CF-EP-CNTF/1CF-EP, i.e., sample 9, 4, 8, 10, the SE curves showed rising trend and then displayed maximum value of about 30 dB with increasing areal density of CNTFs. Detailed analysis of EMI SE of the different CEC composites at 9 GHz was performed and is presented in Figure 3h, emerging a turning point at areal density of 6.8 g/m^2^. This occurrence could be explainable by the formula of EMI materials with *SE_A_* over 10 dB, which can be written as [24,25]:

(1) $$SE\left(dB\right)=SE_{A}+SE_{R}=8.686t{\left(\pi f\mu \sigma \right)}^{1/2}+10log\frac{\sigma }{2\pi f\mu }+39.5$$

where *f* is the incidental frequency of the electromagnetic wave, *t*, *μ* and *σ* are the thickness, permeability and conductivity of the composite, respectively. According to the Formula (1), *SE* variation tendency of CNTFs with different conductivities was fitted in Figure 3i. With increasing conductivity of CNTF (detailed parameters of CNTFs showed in Supplementary Materials Table S2), theoretical SE variation trend of increasingly growth and then basically remaining constant was obtained, wherein conductivity of 4.9 × 10^4^ S/m was the turning point. Therefore, a similar SE growth rule of the CEC materials containing the above CNTFs would be observed, as shown in Figure 3h, due to the similar conductivity changes of the materials with the structure of 1CF-EP-CNTF/1CF-EP, i.e., sample 4 would be a turning point of EMI SE with improvement of CNTF areal density while the structure of them was the same in the X-band. The propagation characteristics of electromagnetic wave almost without transmission was similar with sample 3–7 based on transmission coefficient T of about zero shown in Supplementary Materials Figure S6.

**Figure 4:**
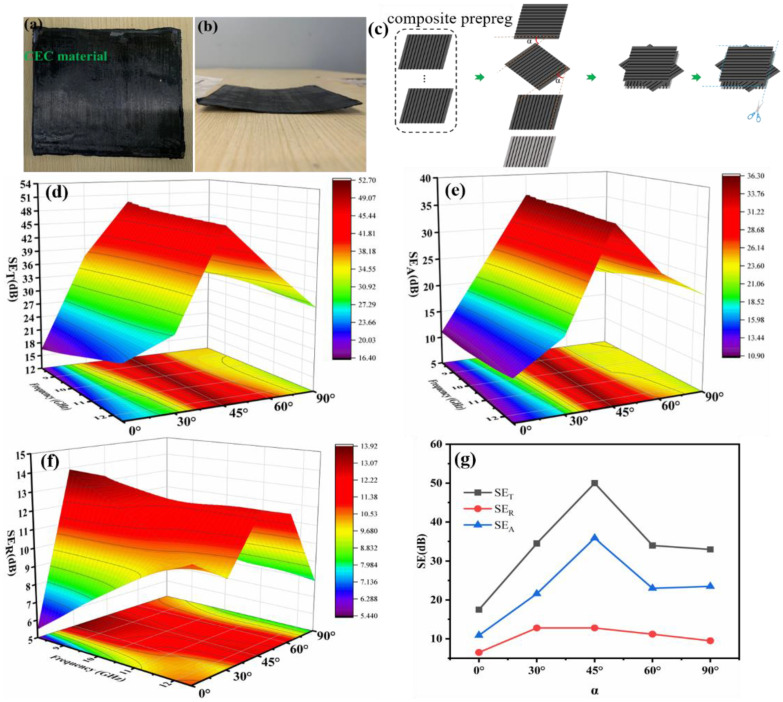
(**a**,**b**) Photographs of the cured CEC sample; (**c**) preparation method of the sample with different α of composite prepregs. Three-dimensional presentation of (**d**) *SE_T_*, (**e**) *SE_A_*, (**f**) *SE_R_* and (**g**) trend of variation of SE at 9 GHz of composites with different α.

The aforementioned samples were fabricated by stacking composite prepregs in one direction and cured via CNTF e-heating, leading to the twisted shapes depicted in Figure 4a,b, although the EMI SE can meet the requirements of some applications. Therefore, the manufacture of samples with different stacked angles α of composite prepregs was conducted to eliminate the defect and evaluate influence of α on the EMI properties as well. The preparation process of uncured samples with structure 1CF-EP-CNTF/1CF-EP-CNTF/1CF-EP-CNTF/1CF-EP with CNTF areal density of 24.8 g/m^2^ and different α was displayed in Figure 4c, then cured samples were obtained via heating process showed in Figure 1b. Figure 4d–f demonstrates the EMI SE of the fabricated CEC composites that exhibit a function of frequency and α from 0° to 90°. Based on the three-dimensional presentation, a similar trend of variation of *SE_T_*, *SE_R_* and *SE_A_* was revealed, additionally, independence of EMI SE on frequency was presented from its 3D projection plots, a peak value of EMI SE for sample with α of 45° appeared in the curve, showing a gradually rise to 50 dB and then decline in value of SE shown in Figure 4g. α could influence the polarization of electric field in CEC composite, the beneficial attenuation of microwave at α of about 45° was caused from a viewpoint of polarization. Besides, EMI properties determined by α was also concerned with electromagnetic parameters, e.g., permittivity ε and permeability μ, which could be subjected to the influence of α in samples [26]. Herein, CEC sample with α of about 45° exhibited more energy loss according to the dielectric loss tangent tanδ described in following section, contributing to the highest EMI SE. 

**Figure 5:**
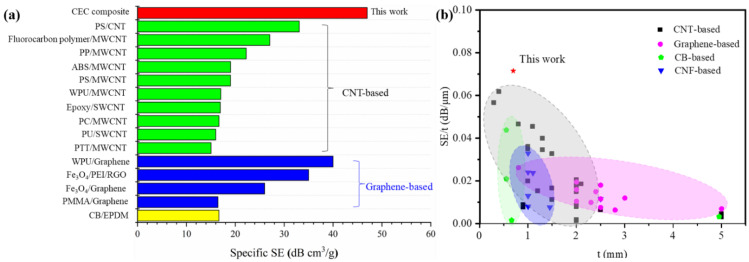
Comparison of EMI shielding performance of different composites: (**a**) specific SE (PS/CNT [27], fluorocarbon polymer/MWCNT [28], PP/MWCNT [25], ABS/MWCNT [29], PS/MWCNT [30], WPU/MWCNT [31], epoxy/SWCNT [32], PC/MWCNT [8], PU/SWCNT [33], PTT/MWCNT [34], WPU/Graphene [35], Fe_3_O_4_/PEI/RGO [36], Fe_3_O_4_/ Graphene [37], PMMA/Graphene [38], CB/EPDM [39]); (**b**) SE/t (the detailed shielding effective and thickness of each shielding composite and references are supplied in Supplementary Materials).

As shown in Supplementary Materials Figure S7, the CEC composites with different α entirely exhibited reflection mechanism and power coefficient T of 0, which disclosed EMI shielding mechanism without influence of α for all samples. As far as shielding ability was concerned, when specific shielding effective of the as-prepared composites was compared with previous reports, we can find that the CEC composites have an obvious advantage over a number of EMI shielding materials shown in Figure 5 (Supplementary Materials Table S3 presented detailed values of SE/t), including many CNT-based, graphene-based, carbon black (CB)-based and carbon nanofiber (CNF)-based shielding materials. The specific SE and SE/t can reach up to at least about 47 dB cm^3^/g and 0.07 dB/μm, respectively. These indicated CEC composites can meet requirements of lightweight and thin materials with higher SE and excellent multifunction, as discussed below. Additionally, the CEC sample with a higher EMI SE of approximately 50 dB can not only be observed but also showed the controllable characteristics of structure and performance by adjusting the number of composite prepreg, CNTF in composite prepreg and α conveniently.

### 3.3. Metamaterial Properties and Shielding Mechanism

**Figure 6:**
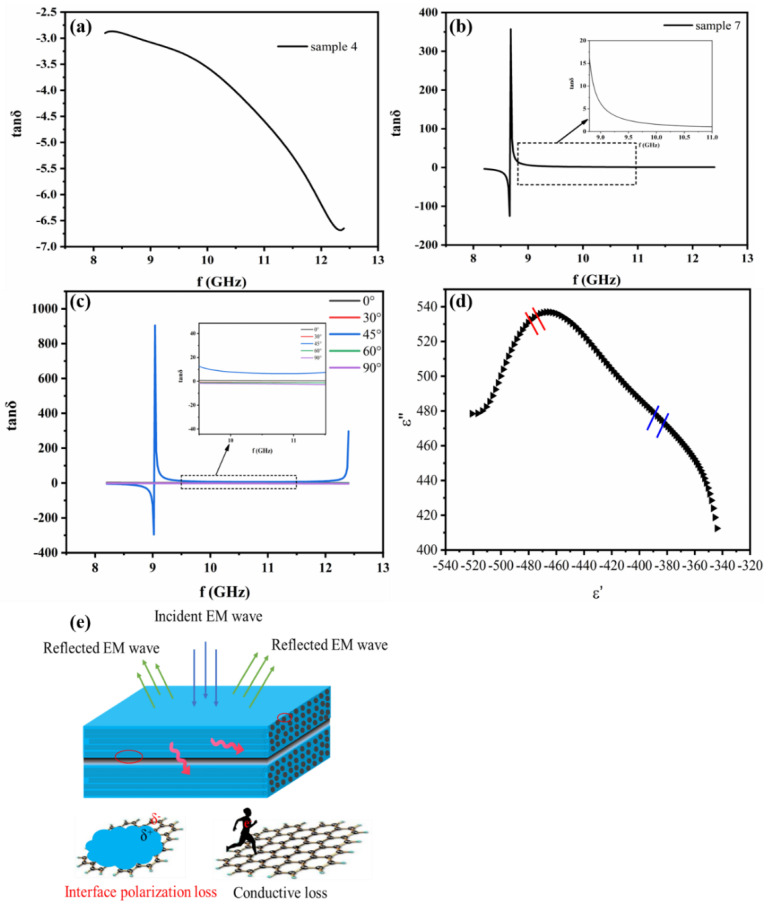
Dielectric loss tangent tanδ of (**a**) samples 4, (**b**) samples 7, and (**c**) samples with different α, (**d**) Cole-Cole semicircle of sample with α of 45° and (**e**) schematic diagram of electromagnetic wave loss mechanism.

Measurements of ε and μ were carried out to further investigate electromagnetic shielding properties of the CEC materials. Supplementary Materials Figure S8 and Figure 6 exhibited real part ε′ and dielectric loss tangent tanδ of the samples in the range of 8.2–12.4 GHz. Adjustable ε′ from positive to negative values were obtained as shown in Figure S8, these materials with negative permittivity were named as a metamaterial, which is an artificially structured material with repeatable structural pattern and is designed for specific shapes and dimensions to affect the EM wave response at a targeted wavelength/frequency [26]. To the best of our knowledge, the study of CEC composite as metamaterials was reported for the first time. The negative ε′ is usually observed in carbon nanotube-based composites due to their metallic nature of filler, leading to the dielectric resonance of the polarization or the plasma like oscillation of the delocalized electrons, which can be described by the Drude model [40]. In the CEC samples, the interface interaction between resin matrix and CF/CNTF would contribute to dielectric resonance of the polarization while the alternative electromagnetic field was supplied, causing the negative ε′. According to the frequency dependencies of permittivity of samples with negative permittivity ε′, as the frequency increases, the ε′ shows an increasing trend. This occurrence can be explained by dielectric relaxation as accessible with the Drude model, which implies that the electric field changes are faster compared to the increasement of frequency, so that the internal electrons cannot keep up with the change in the applied electric field, ultimately leading to the absolute value of the negative permittivity being reduced as described in Supplementary Materials Figure S8. Negative permeability is usually derived from abundantly conductive closed loops, which was not generated by CEC materials owing to the feature of the microstructure without conductive closed loops. As depicted in Figure S9, all obtained CEC samples showed frequency independencies of μ′ with a constant value of approximately 1, and μ″ remained at 0, indicating magnetic loss would not occur in CEC composites. 

In terms of tanδ, two kinds of variation tendencies of it with frequency were observed. The one is the form of sample 4 showing negative value in the whole frequency range depicted in Figure 6a, which could originate from the aforementioned property of metamaterials with negative permittivity of the prepared CEC material. The other is the form of sample 7 showing negative at lower frequencies and then turned positive at transition frequency exhibited in Figure 6b, where sharp negative peak followed by positive peak can be also observed at this frequency and corresponding ε’ switches from negative to positive. The reason contributed to the peaks in the dielectric loss spectra are the interband transition resonance due to the electron transition from bound deeper levels to the conduction band, where the system oscillates with maximum amplitude [40], negative loss appears when the permittivity is negative and can be also observed in previous studies, such as polypropylene/graphene and polyaniline/MWCNT composites [41,42]. Figure 6c presents the tanδ of the CEC materials with various α in the X-band, and it is obvious that the biggest tanδ can be obtained from the composite with α of 45° compared to composites with other α, implying more dielectric loss and higher SE of composite with α of 45° among them [43]. The attenuation mechanism of the electromagnetic wave apart from reflection loss can be explicated by the Cole–Cole curve and schematic diagram of electromagnetic wave loss showed in Figure 6d,e. According to the representative Cole–Cole semicircle, it is clearly seen that there is a semicircle in the CEC composite, demonstrating the presence of Debye relaxation processes along with the interfacial polarization caused by the interfaces between resin and CNTF/CF. Additionally, it is worth pointing out that the conductive loss due to the generation of conductive current from high conductivity of CNT/CF is another factor influencing their dielectric loss, which can be confirmed by the existence of the line tail in the Cole–Cole curve. Therefore, the shielding mechanism of our prepared CEC composites to electromagnetic wave included reflection, interface polarization loss and conductive loss depicted in Figure 6e. 

### 3.4. Multifunctional Properties

**Figure 7:**
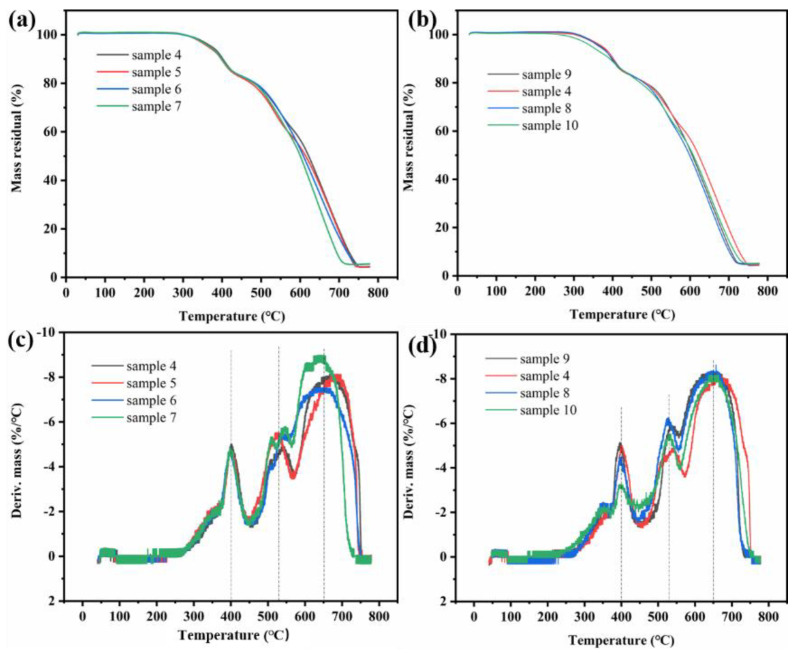
TGA curves of (**a**) sample 4–7 and (**b**) sample 9, 4, 8, 10; DTG curves of (**c**) sample 4–7 and (**d**) sample 9, 4, 8, 10.

As the improvement of shielding materials requirements, materials with multifunctional properties, including high temperature resistance, hydrophobicity and anticorrosion, need to be designed urgently. Based on these, the thermostability detection in air of the as-fabricated shielding materials was carried out firstly. The heating procedure from room temperature to 800 °C with heating rate of 10 °C/min was performed by TGA. The thermostability results of the CEC composites, containing same CNTF and different number of composite prepregs, or same structure with different CNTF, are presented in TGA and DTG curves shown in Figure 7. Three weight decrease stages of the samples were exhibited and was in accordance with the results of peaks in DTG curves, denoting the fastest decomposition temperature of polymer matrix at 400 °C, CF at 525 °C and CNT at 650 °C, respectively. According to the DTG curves, the initial decomposition temperature of the CEC composites can reach up to approximately 300 °C regardless of the structure of composites, indicating the better high temperature resistance of the EMI shielding materials and better structure without damage before initial decomposition temperature. According to previous reports, the as-fabricated CEC composites could be comparable to high temperature resistance carbon-based EMI shielding materials with addition of resin matrices or ceramics particles, such as SiC, SiO_2_, ZnO and CdS, etc. [11], indicating that CEC composites with better thermostability could be devoted to the applications concerning a higher work temperature below the decomposition temperature of them while excellent EMI shielding properties was required. 

**Figure 8:**
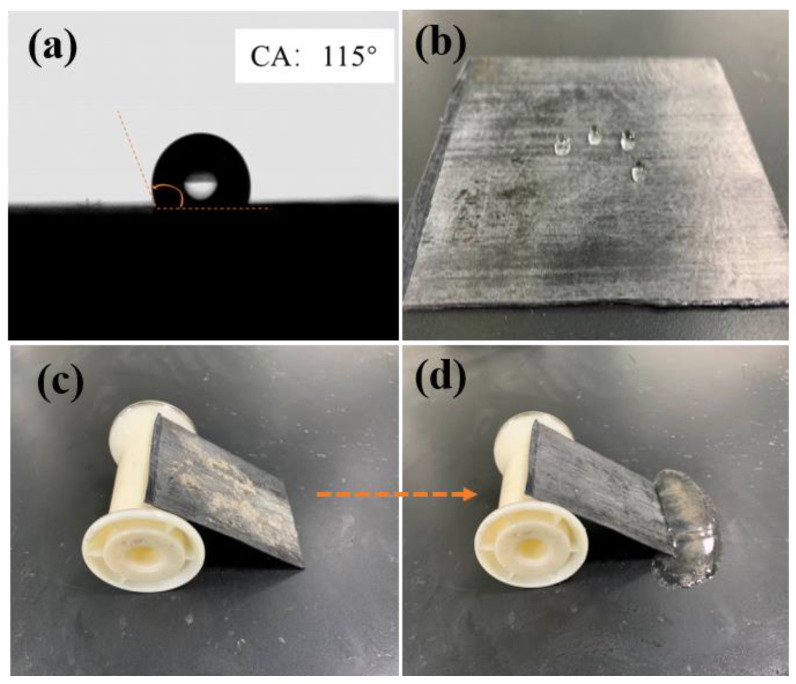
(**a**,**b**) Hydrophobicity and (**c**,**d**) self-cleaning property of CEC composite.

The evaluation of hydrophobicity of the CEC composites was performed subsequently, Figure 8a,b revealed the contact angle (CA) and image of water droplets on the surface of CEC composite, respectively. A higher CA of approximately 115° was observed as shown in Figure 8a, and water droplets approximately exhibited spherical form, which could easily slide off the surface of CEC composite (a video about hydrophobicity of the composite was provided). Thus, the excellent self-cleaning ability of the CEC composite was exhibited due to the hydrophobicity, as displayed in Figure 8c,d. The much hydrophobic benzene ring structure in the material could be responsible for the self-cleaning effect while the EMI shielding composite was contaminated. 

**Figure 9:**
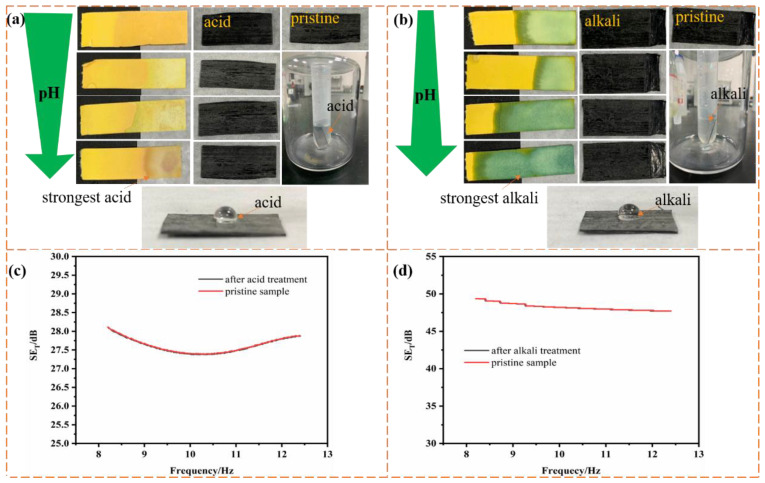
(**a**,**b**) Digital images and (**c**,**d**) electromagnetic shielding efficiency of samples 8, 14 before and after acid-alkali treatment.

Acid and alkali resistance of materials, especially shielding materials without metal modification, is highly desirable when they are applied in extremely stringent conditions. Therefore, the acid and alkali resistance of the as-prepared CEC composites in the solutions with different acid and alkali concentrations was investigated. Figure 9a,b depicts the surface state of the samples 8 and 14 after being immersed in acid and alkali solutions for about 30 min, respectively. As we can clearly see from the digital images, the obvious corrosion effect on the surface of CEC material would not be introduced by acid and alkali solution compared with pristine samples, regardless of the concentration of acid and alkali solution, in addition, Figure 9a,b showed that CEC composites were incompatible with acid and alkaline solutions with higher CA of over 90°. Figure 9c,d demonstrated the variation tendency of shielding efficiency of samples 8 and 14 in X-band before and after the strongest acid and alkali solution treatment for about 30 min, respectively, it is obvious that the electromagnetic shielding efficiency of the material did not exhibit significant difference after the acid and alkali treatment in contrast with pristine samples, which may be attributable to the better acid and alkali resistance of cross-linking network and benzene ring structure of epoxy resin matrix, CF reinforcement and CNT in the composites. As a result, the excellent self-cleaning and corrosion resistance of the CEC composites with metal-free components and function as structural laminates were obtained, promoting their application in the field of electromagnetic shielding, compared to previous reports of anticorrosion EMI shielding sponges, textiles and composite modified by Ag or Ni/W/P with disadvantages in weight and cost [13,44,45]. 

## 4. Conclusions

In conclusion, multifunctional nanocomposite with composite prepregs were successfully manufactured via state-of-the-art CNTF e-heating. The results indicated that CEC material with reflection loss, interface polarization loss and conductive loss mechanism could exhibit superior EMI properties over that of lots of previous reported CNT-based, graphene-based, CB-based and CNF-based shielding materials, and EMI shielding properties of them can be controlled by adjusting composite prepreg, including stacking number and stacking angle of composite prepregs, and CNTF. Importantly, multifunctional properties, especially corrosion resistance of them without metal modification were disclosed, along with thermostability of initial degradation temperature at 300 °C and the fastest decomposition temperature at 400 °C and excellent hydrophobicity, suggesting huge potential of the application in EMI shielding field.

## Figures and Tables

**Table 1:** Presentations of sample 1~15.

Areal Density of CNTF	Samples *	Name
6.8 g/m^2^	(1CF-EP-CNTF/1CF-EP) _oven/0°_	sample 1
	(1CF-EP-CNTF/1CF-EP) _hot press/0°_	sample 2
	(1CF-EP-CNTF/1CF-EP) _e-heating/0°_	sample 4
	(1CF-EP/1CF-EP) _e-heating/0°_	sample 3
	(1CF-EP-CNTF/1CF-EP-CNTF/1CF-EP) _e-heating/0°_	sample 5
	(1CF-EP/1CF-EP-CNTF/1CF-EP-CNTF/1CF-EP) _e-heating/0°_	sample 6
	(1CF-EP-CNTF/1CF-EP-CNTF) _e-heating/0°_	sample 7
12.5 g/m^2^	(1CF-EP-CNTF/1CF-EP) _e-heating/0°_	sample 8
5.48 g/m^2^	(1CF-EP-CNTF/1CF-EP) _e-heating/0°_	sample 9
24.8 g/m^2^	(1CF-EP-CNTF/1CF-EP) _e-heating/0°_	sample 10
	(1CF-EP-CNTF/1CF-EP-CNTF/1CF-EP-CNTF/1CF-EP) _e-heating/0°_	sample 11
	(1CF-EP-CNTF/1CF-EP-CNTF/1CF-EP-CNTF/1CF-EP) _e-heating/30°_	sample 12
	(1CF-EP-CNTF/1CF-EP-CNTF/1CF-EP-CNTF/1CF-EP) _e-heating/45°_	sample 13
	(1CF-EP-CNTF/1CF-EP-CNTF/1CF-EP-CNTF/1CF-EP) _e-heating/60°_	sample 14
	(1CF-EP-CNTF/1CF-EP-CNTF/1CF-EP-CNTF/1CF-EP) _e-heating/90°_	sample 15

* Oven/0°, hot press/0°, e-heating/0° denote that samples were heated by oven, hot press and e-heating, respectively and composite prepregs were stacked up in one direction (stacked angle of composite prepreg is 0°).

## Data Availability

Not applicable.

## Supplementary Materials

The following are available online at https://www.mdpi.com/article/10.3390/nano11113155/s1, Figure S1: Microscope images of (a) sample 1, (b) sample 2, (c) sample 4 heated by oven, hot press and CNTF e-heating, respectively. Figure S2: Microscope images of (a) sample 3, (b) sample 4, (c) sample 5 and (d) sample 7. Figure S3: Microscope images of (a) sample 9, (b) sample 4, (c) sample 8 and (d) sample 10. Figure S4: DSC curves of (a) uncured pristine prepreg and sample 1, 2, 4 (oven, hot press and e-heating, respectively), (b) uncured pristine prepreg and sample 3-7 (c) uncured pristine prepreg and sample 9, 4, 8, 10. Figure S5: Power coefficients R, T, A of (a) sample 3, (b) sample 4 and (c) sample 5 (d) sample 6, (e) sample 7. Figure S6: Power coefficients R, T, A of (a) sample 9, (b) sample 4, (c) sample 8 (d) sample 10. Figure S7: Power coefficients R, T, A of (a) sample 11, (b) sample 12, (c) sample 13, (d) sample 14 and (e) sample 15. Figure S8: Real part of permittivity ε’ of (a) samples 1, 2, 4, (b) samples 3-7, (c) samples 9, 4, 8, 10 with gradually enhanced areal density of CNTF, (d) samples 11-15. Figure S9: Real part of permeability μ′ of (a) samples 1, 2, 4, (b) samples 3-7, (c) samples 9, 4, 8, 10, (d) samples 11-15; imaginary part μ″ of (e) samples 1, 2, 4, (f) samples 3-7, (g) samples 9, 4, 8, 10, (h) samples 11-15. Table S1: Curing degree of the sample 1-10. Table S2: Parameters of CNTF in composite prepreg. Table S3: Comparison of EMI shielding performance of different composites. Video S1: Hydrophobicity nanocomposite laminate.
