# Supplementary material for: EMI Shielding Nanocomposite Laminates with High Temperature Resistance, Hydrophobicity and Anticorrosion Properties
Source: Nanomaterials (Basel). 2021 Nov 22;11(11):3155. doi: 10.3390/nano11113155 (PMC8621499; doi:10.3390/nano11113155)
Supplement: Supplementary file 1 [file nanomaterials-11-03155-s001.zip › nanomaterials-1465651-Supplementary Material.pdf]

Supplementary Materials

EMI Shielding Nanocomposite Laminates with High Temperature Resistance, Hydrophobicity and Anticorrosion Properties

Shaojun Wu [1,2], Zhiyong Zhao [3], Hongliang Hou [2,*] and Xiang Xue [1,*]

1 School of Materials Science and Engineering, Harbin Institute of Technology, Harbin 150001, China; wusj0303@163.com
2 AVIC Manufacturing Technology Institute, Beijing 100024, China
3 School of Mechanical, Electrical and Information Engineering, Shandong University (Weihai), Weihai 264209, China; zhaozhy@sdu.edu.cn
* Correspondence: hou_hl@163.com (H.H.); xxue@hit.edu.cn (X.X.)

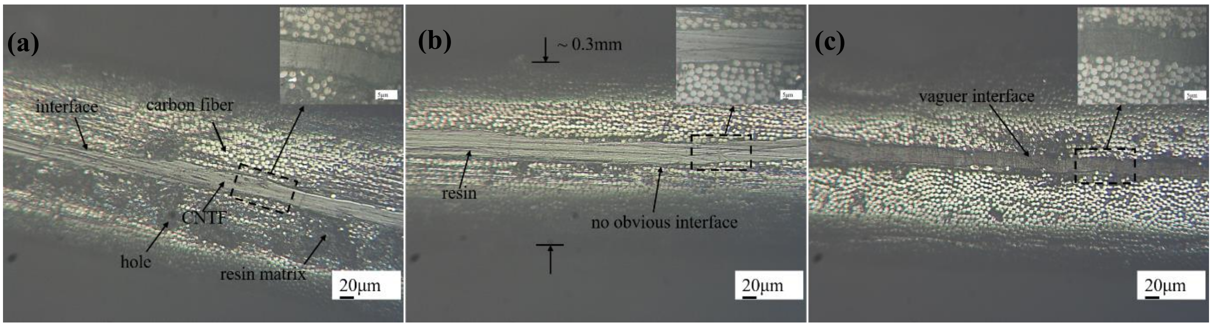

Figure S1. Microscope images of (**a**) sample 1, (**b**) sample 2, (**c**) sample 4 heated by oven, hot press and CNTF e-heating, respectively.

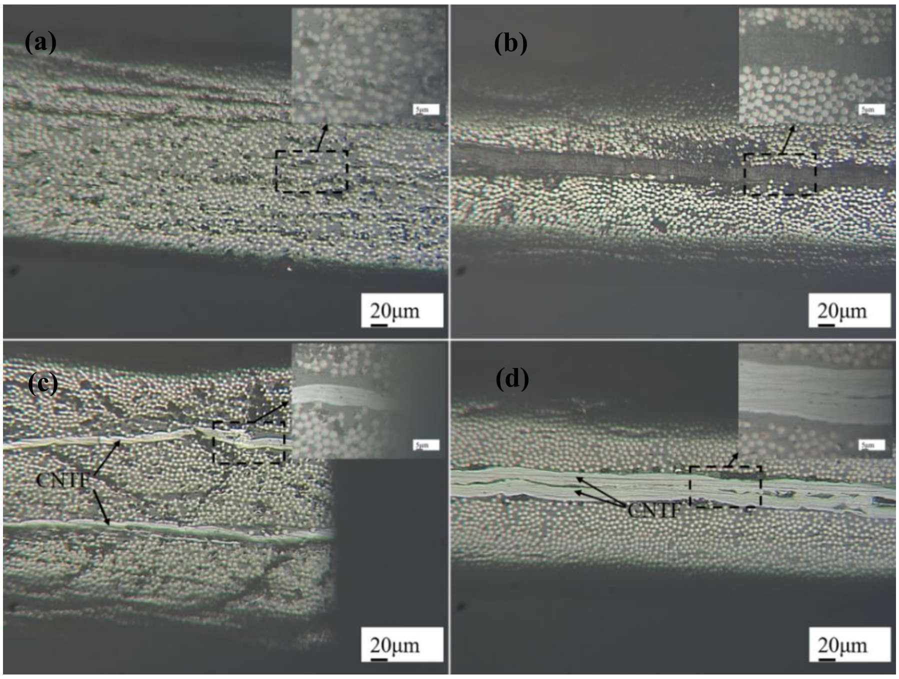

Figure S2. Microscope images of (**a**) sample 3, (**b**) sample 4, (**c**) sample 5 and (**d**) sample 7.

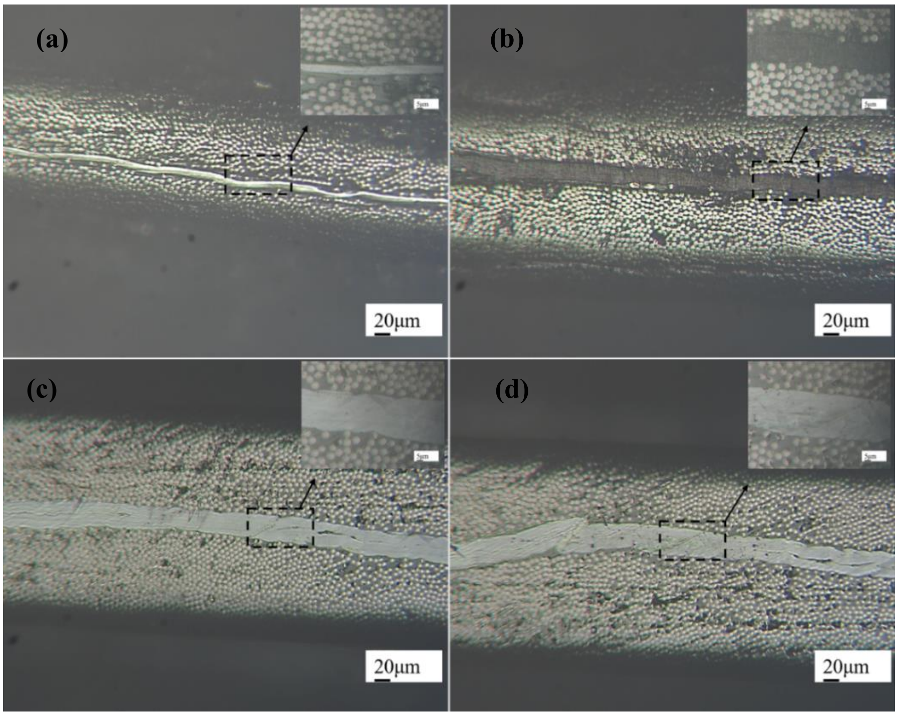

Figure S3. Microscope images of (**a**) sample 9, (**b**) sample 4, (**c**) sample 8 and (**d**) sample 10.

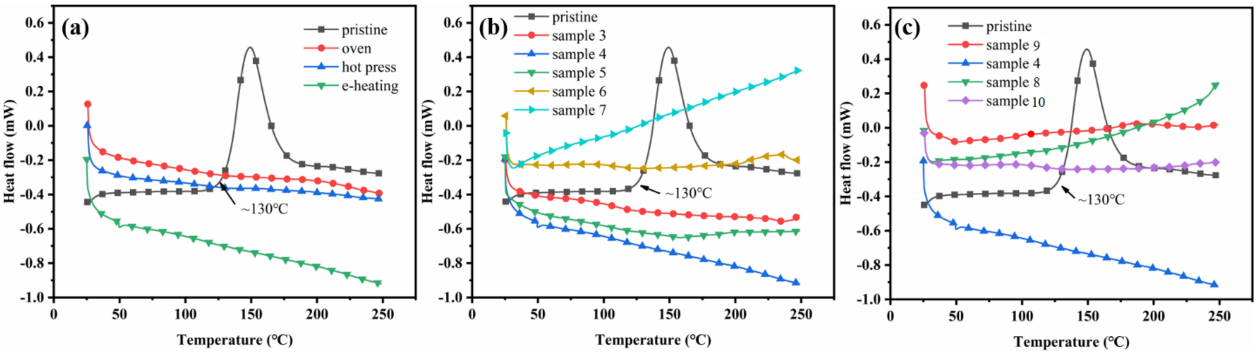

Figure S4. DSC curves of (**a**) uncured pristine prepreg and sample 1, 2, 4 (oven, hot press and e-heating, respectively), (**b**) uncured pristine prepreg and sample 3-7 (**c**) uncured pristine prepreg and sample 9, 4, 8, 10.

Table S1. Curing degree of the sample 1-10.

Samples	1 (oven)	2 (hot press)	4 (e-heating)	3	5
Curing degree	99.9%	99.5%	99.8%	99.9%	99.9%
Samples	6	7	8	9	10
Curing degree	99.8%	99.2%	99.8%	99.9%	99.6%

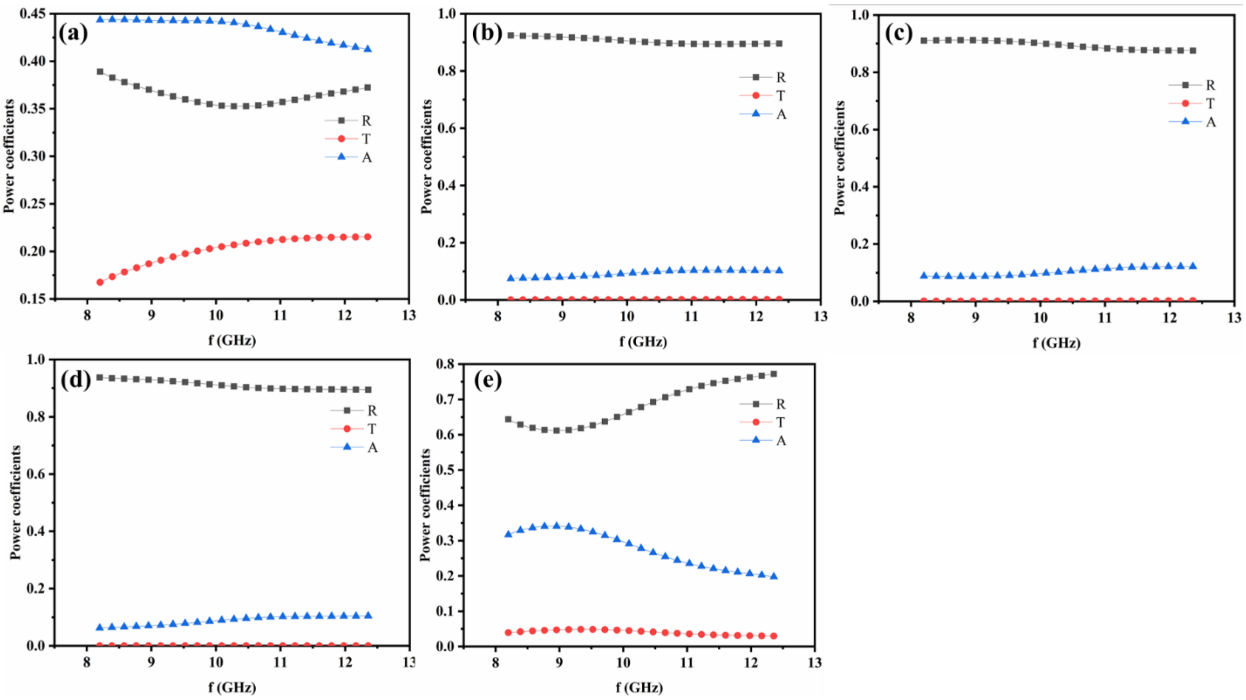

Figure S5. Power coefficients R, T, A of (**a**) sample 3, (**b**) sample 4 and (**c**) sample 5 (**d**) sample 6, (**e**) sample 7.

Table S2. Parameters of CNTF in composite prepreg.

sample	9	4	8	10
areal density (g/m^2)	5.48	6.8	12.5	24.8
t (μm)	18.7	23.37	34	60
R (Ω/□)	2.46	0.88	0.55	0.3
σ (S/m)	2.2×10^4	4.9×10^4	5.3×10^4	5.6×10^4

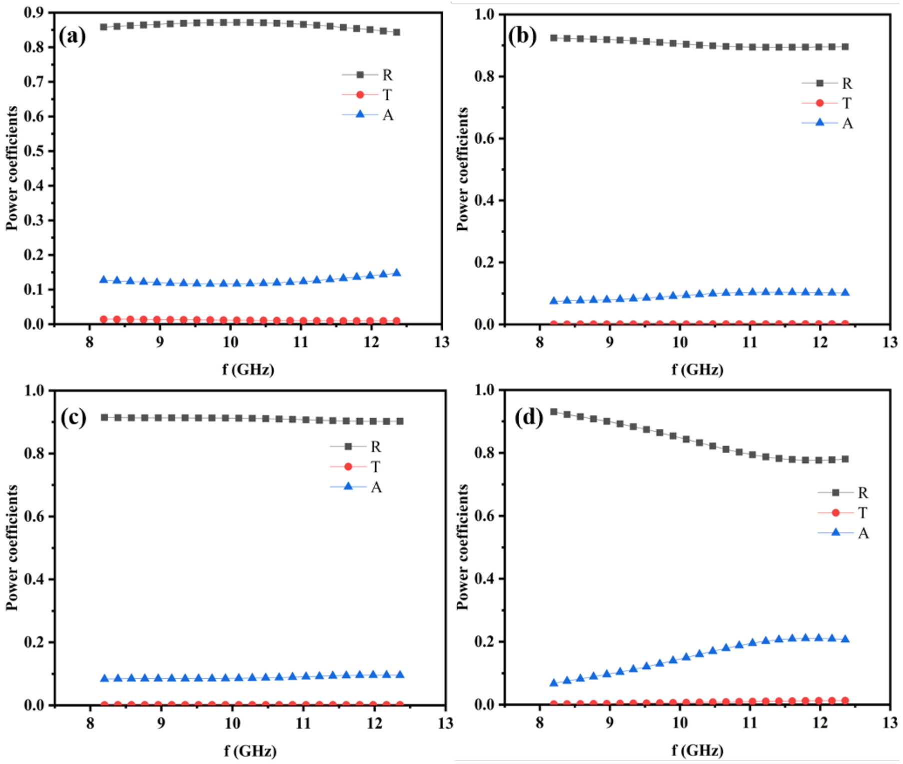

Figure S6. Power coefficients R, T, A of (**a**) sample 9, (**b**) sample 4, (**c**) sample 8 (**d**) sample 10.

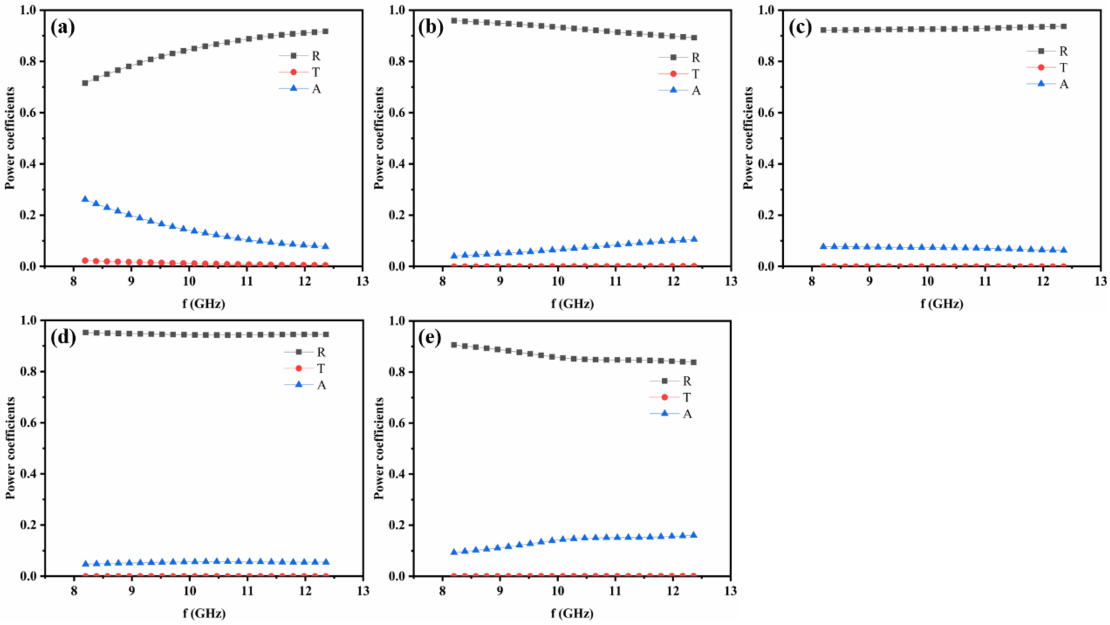

Figure S7. Power coefficients R, T, A of (**a**) sample 11, (**b**) sample 12, (**c**) sample 13, (**d**) sample 14 and (**e**) sample 15.

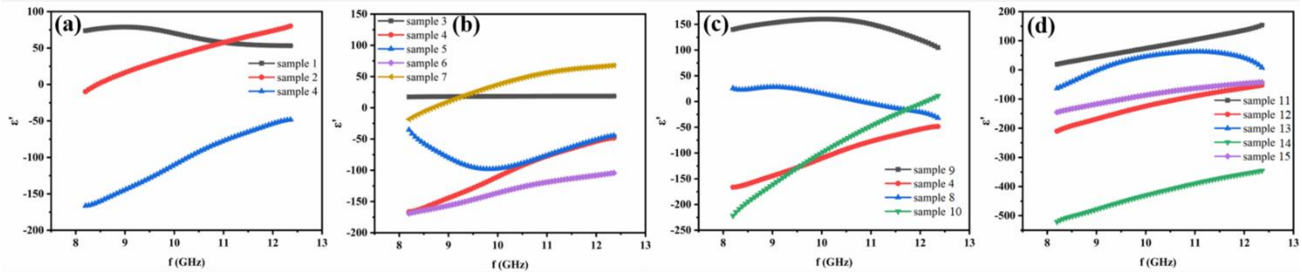

Figure S8. Real part of permittivity ε' of (**a**) samples 1, 2, 4, (**b**) samples 3-7, (**c**) samples 9, 4, 8, 10 with gradually enhanced areal density of CNTF, (**d**) samples 11-15.

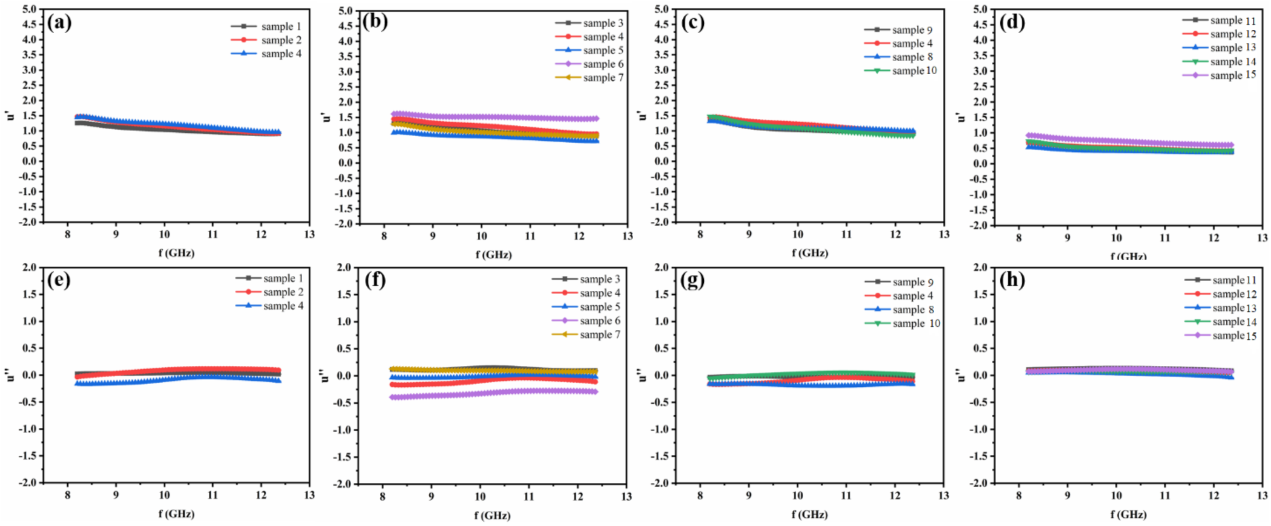

Figure S9. Real part of permeability μ' of (**a**) samples 1, 2, 4, (**b**) samples 3-7, (**c**) samples 9, 4, 8, 10, (**d**) samples 11-15; imaginary part μ'' of (**e**) samples 1, 2, 4, (**f**) samples 3-7, (**g**) samples 9, 4, 8, 10, (**h**) samples 11-15.

Table S3. Comparison of EMI shielding performance of different composites.

Type	Filler	Matrix*	Thickness (mm)	SSE(dB/μm)	References
CNT	MWCNT	PP	1	0.035	[1]
	MWCNT	CPE	1	0.036	[2]
	MWCNT	CPE	5	0.0044	[3]
	MWCNT	PLLA	1.3	0.0346	[4]
	MWCNT	iPP	1.3	0.04	[4]
	MWCNT	PLLA/PCL	1.5	0.0113	[5]
	SWCNT (High aspect ratio)	Epoxy	1.5	0.0327	[6]
	CNT (Long length)	Epoxy	2.5	0.0064	[7]
	SWCNT	Epoxy	1	0.02	[8]
	SWCNT	PS	1.2	0.0154	[9]
	MWCNT	WPU	1	0.0211	[10]
	MWCNT	PS	2	0.015	[11]
	MWCNT	PC	2.1	0.0186	[12]
	MWCNT	ABS	1.1	0.0455	[13]
	MWCNT	PA	1.5	0.0167	[14]
	MWCNT	SEBS	2	0.01535	[15]
	MWCNT	PU	2	0.0206	[16]
	MWCNT	PU	2	0.008	[17]
	MWCNT	PS	2	0.0086	[18]
	MWCNT	PE	2	0.0015	[19]
	MWCNT	PTT	2	0.0019	[20]
	MWCNT	PS	1	0.02	[21]
	MWCNT	TPU	2	0.01765	[22]
	MWCNT	PS	0.3	0.0567	[23]
	MWCNT	PVDF	2	0.0184	[24]
	MWCNT	WPU	0.4	0.06175	[25]
	MWCNT	PC	0.9	0.0078	[26]
	MWCNT	PVDF	0.9	0.0089	[27]
	MWCNT	PVDF/PC	5	0.0032	[27]
	MWCNT	PVDF/ABS	5	0.0046	[28]
	MWCNT	TPU	0.8	0.0466	[29]
CNF	CNF	EMA	2.5	0.0116	[30]
	CNF	ABS	1.1	0.0237	[31]
	CNF	PI	1	0.013	[32]
	CNF	CPE	1	0.024	[33]
	VGCNF	PS	1	0.008	[34]
	CB/CNF	CPE	1	0.0329	[35]
	VGCNF	LCP	1.45	0.0076	[36]
	CNF	PS	1	0.0129	[37]
CB	CB	ABS	1.1	0.0018	[13]
	CB	CPE	1	0.0212	[38]
	CB	SEBS	5	0.00352	[39]
	CB	Epoxy	1	0.044	[40]
Graphene	RGO	PI	0.8	0.02625	[41]
	RGO	PEI	2.3	0.01	[42]
	IRGO	EMA	5	0.007	[43]
	RGO	PS	2.5	0.0116	[44]
	RGO	PS	2.5	0.01804	[45]
	CVD graphene	PDMS	3	0.012	Z[46]
	Graphene	PMMA	2.5	0.0076	Z[47]
	Functionalized graphene	PS	2.5	0.0116	[48]
	graphene	PU	2	0.016	[49]
	rGO	MWCNT	2.4	0.015	[50]

	Multilayer graphene	PVA	2	0.0195	[51]
	RGO	Epoxy	2	0.0105	[52]
	RGO	PS	2.8	0.0064	[53]

* PP- Polypropylene, CPE- Chlorinated polyethylene, PLLA- Poly(l-lactide), PCL-Poly(e-caprolactone, iPP- Isotactic polypropylene, TPU- Thermoplastic polyurethane, PS-Polystyrene, PU- polyurethane, BR- Butyl rubber, EVA- Ethylene vinyl acetate, PMMA-Polymethylmethacrylate, WPU- Waterborne polyurethane, PC-Polycarbonate, ABS- Acrylonitrile butadiene, PA- Polyamides, SEBS- Styrene-ethylene butylene styrene, PE- Polyethylene, PTT- Poly (trimethylene terephthalate), CPE- Chlorinated polyethylene, PVDF-Polyvinylidene fluoride, LCP-liquid crystal polymer. PDMS- polydimethylsiloxane, EMA- Ethylene methacrylate, PI- Polyimide, PEI- Polyethylenimine, PVA-Poly (vinyl alcohol), RGO-Reduced graphene oxide, IRGO- *insitu* reduced graphene oxide

References

[1] Al-Saleh MH, Sundararaj U. Electromagnetic interference shielding mechanisms of CNT/polymer composites. Carbon. 2009;47(7):1738-46.

[2] Mondal S, Das P, Ganguly S, Ravindren R, Remanan S, Bhawal P, et al. Thermal-air ageing treatment on mechanical, electrical, and electromagnetic interference shielding properties of lightweight carbon nanotube based polymer nanocomposites. Composites Part A: Applied Science and Manufacturing. 2018;107:447-60.

[3] Basuli U, Chattopadhyay S, Nah C, Chaki TK. Electrical properties and electromagnetic interference shielding effectiveness of multiwalled carbon nanotubes-reinforced EMA nanocomposites. Polymer composites. 2012;33(6):897-903.

[4] Shi Y-D, Li J, Tan Y-J, Chen Y-F, Wang M. Percolation behavior of electromagnetic interference shielding in polymer/multi-walled carbon nanotube nanocomposites. Composites Science and Technology. 2019;170:70-6.

[5] Zhang K, Yu H-O, Shi Y-D, Chen Y-F, Zeng J-B, Guo J, et al. Morphological regulation improved electrical conductivity and electromagnetic interference shielding in poly (L-lactide)/poly (ε-caprolactone)/carbon nanotube nanocomposites via constructing stereocomplex crystallites. Journal of Materials Chemistry C. 2017;5(11):2807-17.

[6] Li N, Huang Y, Du F, He X, Lin X, Gao H, et al. Electromagnetic interference (EMI) shielding of single-walled carbon nanotube epoxy composites. Nano Letters. 2006;6(6):1141-5.

[7] Singh B, Saini K, Choudhary V, Teotia S, Pande S, Saini P, et al. Effect of length of carbon nanotubes on electromagnetic interference shielding and mechanical properties of their reinforced epoxy composites. Journal of Nanoparticle Research. 2014;16(1):2161.

[8] Li N, Huang Y, Du F, He X, Lin X, Gao H, et al. Electromagnetic interference (EMI) shielding of single-walled carbon nanotube epoxy composites. Nano Letters. 2006;6(6):1141-5.

[9] Yang Y, Gupta MC, Dudley KL, Lawrence RW. Novel carbon nanotube− polystyrene foam composites for electromagnetic interference shielding. Nano Letters. 2005;5(11):2131-4.

[10] Zeng Z, Jin H, Chen M, Li W, Zhou L, Zhang Z. Lightweight and anisotropic porous MWCNT/WPU composites for ultrahigh performance electromagnetic interference shielding. Advanced Functional Materials. 2016;26(2):303-10.

[12] Arjmand M, Apperley T, Okoniewski M, Sundararaj U. Comparative study of electromagnetic interference shielding properties of injection molded versus compression molded multi-walled carbon nanotube/polystyrene composites. Carbon. 2012;50(14):5126-34.

[13] Pande S, Chaudhary A, Patel D, Singh BP, Mathur RB. Mechanical and electrical properties of multiwall carbon nanotube/polycarbonate composites for electrostatic discharge and electromagnetic interference shielding applications. RSC Advances. 2014;4(27):13839-49.

[14] Al-Saleh MH, Saadeh WH, Sundararaj U. EMI shielding effectiveness of carbon based nanostructured polymeric materials: a comparative study. Carbon. 2013;60:146-56.

[15] Li Y, Chen C, Zhang S, Ni Y, Huang J. Electrical conductivity and electromagnetic interference shielding characteristics of multiwalled carbon nanotube filled polyacrylate composite films. Applied Surface Science. 2008;254(18):5766-71.

[16] Kuester S, Barra GM, Ferreira Jr JC, Soares BG, Demarquette NR. Electromagnetic interference shielding and electrical properties of nanocomposites based on poly (styrene-b-ethylene-ran-butylene-b-styrene) and carbon nanotubes. European Polymer Journal. 2016;77:43-53.

[17] Wang T, Yu W-C, Sun W-J, Jia L-C, Gao J-F, Tang J-H, et al. Healable polyurethane/carbon nanotube composite with segregated structure for efficient electromagnetic interference shielding. Composites Science and Technology. 2020;200:108446.

[18] Ramôa SD, Barra GM, Oliveira RV, de Oliveira MG, Cossa M, Soares BG. Electrical, rheological and electromagnetic interference shielding properties of thermoplastic polyurethane/carbon nanotube composites. Polymer International. 2013;62(10):1477-84.

[19] Mahmoodi M, Arjmand M, Sundararaj U, Park S. The electrical conductivity and electromagnetic interference shielding of injection molded multi-walled carbon nanotube/polystyrene composites. Carbon. 2012;50(4):1455-64.

[20] Yim Y-J, Park S-J. Electromagnetic interference shielding effectiveness of high-density polyethylene composites reinforced with multi-walled carbon nanotubes. Journal of Industrial and Engineering Chemistry. 2015;21:155-7.

[21] Gupta A, Choudhary V. Electromagnetic interference shielding behavior of poly (trimethylene terephthalate)/multi-walled carbon nanotube composites. Composites Science and Technology. 2011;71(13):1563-8.

[22] Yang Y, Gupta MC, Dudley KL. Towards cost-efficient EMI shielding materials using carbon nanostructure-based nanocomposites. Nanotechnology. 2007;18(34):345701.
[23] Feng D, Xu D, Wang Q, Liu P. Highly stretchable electromagnetic interference (EMI) shielding segregated polyurethane/carbon nanotube composites fabricated by microwave selective sintering. Journal of Materials Chemistry C. 2019;7(26):7938-46.
[24] Mathur R, Pande S, Singh B, Dhami T. Electrical and mechanical properties of multi-walled carbon nanotubes reinforced PMMA and PS composites. Polymer Composites. 2008;29(7):717-27.
[25] Yu W-C, Wang T, Zhang G-Q, Wang Z-G, Yin H-M, Yan D-X, et al. Largely enhanced mechanical property of segregated carbon nanotube/poly(vinylidene fluoride) composites with high electromagnetic interference shielding performance. Composites Science and Technology. 2018;167:260-7.
[26] Li H, Yuan D, Li P, He C. High conductive and mechanical robust carbon nanotubes/waterborne polyurethane composite films for efficient electromagnetic interference shielding. Composites Part A: Applied Science and Manufacturing. 2019;121:411-7.
[27] Biswas S, Arief I, Panja SS, Bose S. Absorption-dominated electromagnetic wave suppressor derived from ferrite-doped cross-linked graphene framework and conducting carbon. ACS Applied Materials & Interfaces. 2017;9(3):3030-9.
[28] Kar GP, Biswas S, Rohini R, Bose S. Tailoring the dispersion of multiwall carbon nanotubes in co-continuous PVDF/ABS blends to design materials with enhanced electromagnetic interference shielding. Journal of Materials Chemistry A. 2015;3(15):7974-85.
[29] Bs B, Sm A, ML B , et al. Flexible thermoplastic polyurethane-carbon nanotube composites for electromagnetic interference shielding and thermal management. Chemical Engineering Journal, 2021; 418.
[30] Bhawal P, Ganguly S, Das TK, Mondal S, Nayak L, Das NC. A comparative study of physico-mechanical and electrical properties of polymer-carbon nanofiber in wet and melt mixing methods. Materials Science and Engineering: B. 2019;245:95-106.
[31] Al-Saleh MH, Saadeh WH, Sundararaj U. EMI shielding effectiveness of carbon based nanostructured polymeric materials: a comparative study. Carbon. 2013;60:146-56.
[32] Nayak L, Chaki TK, Khastgir D. Electrical percolation behavior and electromagnetic shielding effectiveness of polyimide nanocomposites filled with carbon nanofibers. Journal of Applied Polymer Science. 2014;131(24).
[33] Mondal S, Nayak L, Rahaman M, Aldalbahi A, Chaki TK, Khastgir D, et al. An effective strategy to enhance mechanical, electrical, and electromagnetic shielding effectiveness of chlorinated polyethylene-carbon nanofiber nanocomposites. Composites Part B: Engineering. 2017;109:155-69.
[34] Yang Y, Gupta M C, Dudley K L, et al. A comparative study of EMI shielding properties of carbon nanofiber and multi-walled carbon nanotube filled polymer composites. Journal of Nanoscience & Nanotechnology, 2005, 5(6):927.
[35] Mondal S, Ravindren R, Bhawal P, Shin B, Ganguly S, Nah C, et al. Combination effect of carbon nanofiber and ketjen carbon black hybrid nanofillers on mechanical, electrical, and electromagnetic interference shielding properties of chlorinated polyethylene nanocomposites. Composites Part B: Engineering. 2020;197:108071.
[36] Yang S, Lozano K, Lomeli A, et al. Electromagnetic interference shielding effectiveness of carbon nanofiber/LCP composites. Composites Part A: Applied Science & Manufacturing, 2005, 36(5):691-697.
[37] Yang Y, Gupta M C, Dudley K L. Towards cost-efficient EMI shielding materials using carbon nanostructure-based nanocomposites. Nanotechnology, 2007, 18(34):345701.
[38] Mondal S, Ganguly S, Rahaman M, Aldalbahi A, Chaki TK, Khastgir D, et al. A strategy to achieve enhanced electromagnetic interference shielding at low concentration with a new generation of conductive carbon black in a chlorinated polyethylene elastomeric matrix. Physical Chemistry Chemical Physics. 2016;18(35):24591-9.
[39] Kuester S, Merlini C, Barra GM, Ferreira JC, Lucas A, de Souza AC, et al. Processing and characterization of conductive composites based on poly (styrene-b-ethylene-ran-butylene-b-styrene)(SEBS) and carbon additives: A comparative study of expanded graphite and carbon black. Composites Part B: Engineering. 2016;84:236-47.
[40] Aal NA, El-Tantawy F, Al-Hajry A, Bououdina M. New antistatic charge and electromagnetic shielding effectiveness from conductive epoxy resin/plasticized carbon black composites. Polymer Composites. 2008;29(2):125-32.
[41] Li Y, Pei X, Shen B, Zhai W, Zhang L, Zheng W. Polyimide/graphene composite foam sheets with ultrahigh thermostability for electromagnetic interference shielding. RSC Advances. 2015;5(31):24342-51.
[42] Ling J, Zhai W, Feng W, Shen B, Zhang J, Zheng Wg, et al. Facile preparation of lightweight microcellular polyetherimide/graphene composite foams for electromagnetic interference shielding. ACS Applied Materials & Interfaces. 2013;5(7):2677-84.
[43] Bhawal P, Ganguly S, Das TK, Mondal S, Choudhury S, Das N. Superior electromagnetic interference shielding effectiveness and electro-mechanical properties of EMA-IRGO nanocomposites through the in-situ reduction of GO from melt blended EMA-GO composites. Composites Part B: Engineering. 2018;134:46-60.
[44] Yan D-X, Ren P-G, Pang H, Fu Q, Yang M-B, Li Z-M. Efficient electromagnetic interference shielding of lightweight graphene/polystyrene composite. Journal of Materials Chemistry. 2012;22(36):18772-4.
[45] Yan DX, Pang H, Li B, Vajtai R, Xu L, Ren PG, et al. Structured reduced graphene oxide/polymer composites for ultra-efficient electromagnetic interference shielding. Advanced Functional Materials. 2015;25(4):559-66.
[46] Chen Z, Xu C, Ma C, et al. Lightweight and Flexible Graphene Foam Composites for High-Performance Electromagnetic Interference Shielding. Advanced Materials, 2013, 25(9):1296-1300.
[47] Zhang H B, Yan Q, Zheng W G, et al. Tough graphene-polymer microcellular foams for electromagnetic interference shielding. ACS Applied Materials & Interfaces, 2011, 3(3):918-924.
[48] Ding-Xiang, Yan, Peng-Gang, et al. Efficient electromagnetic interference shielding of lightweight graphene/polystyrene composite. Journal of Materials Chemistry, 2012.

[49] Hsiao S T, Ma C, Tien H W, et al. Using a non-covalent modification to prepare a high electromagnetic interference shielding performance graphene nanosheet/water-borne polyurethane composite. Carbon, 2013, 60:57-66.
[50] Singh A P, Mishra M, Hashim D P, et al. Probing the engineered sandwich network of vertically aligned carbon nanotube–reduced graphene oxide composites for high performance electromagnetic interference shielding applications. Carbon, 2015.
[51] Joseph J, Sharma A, Sahoo B, et al. PVA/MLG/MWCNT hybrid composites for X-band EMI shielding-Study of mechanical, electrical, thermal and tribological properties - ScienceDirect. Materials Today Communications.
[52] Liang J, Wang Y, Huang Y, et al. Electromagnetic interference shielding of graphene/epoxy composites. Carbon, 2009, 47(3):922-925.
[53] Li C, Yang G, Deng H, et al. The preparation and properties of polystyrene/functionalized graphene nanocomposite foams using supercritical carbon dioxide. Polymer International, 2013, 62(7):1077-1084.
